# Mental health during the COVID-19 pandemic: Stress and strain profiles in the German population

**DOI:** 10.3389/fpubh.2023.990407

**Published:** 2023-04-11

**Authors:** Vincent M. E. L. Nin, Gerd-Dieter Willmund, Stefanie M. Jungmann, Gordon J. G. Asmundson, Martina Piefke

**Affiliations:** ^1^Neurobiology and Genetics of Behavior, Department of Psychology and Psychotherapy, Witten/Herdecke University, Witten, Germany; ^2^German Center for Military Mental Health, Military Hospital Berlin, Berlin, Germany; ^3^Department of Clinical Psychology, Psychotherapy, and Experimental Psychopathology, Johannes Gutenberg-University Mainz, Mainz, Germany; ^4^Department of Psychology, University of Regina, Regina, SK, Canada

**Keywords:** pandemic-related stress, SARS-CoV-2 infection, quality of life, phases of pandemic, personality, gender, extraversion, neuroticism

## Abstract

Clinical observations indicate that people frequently display stress-related behavior during the COVID-19 pandemic. Although numerous studies have been published concerning pandemic-related psychological distress, systematic data on the interrelationships between stress sensitivity, personality, and behavioral characteristics of people are still lacking. In the present cross-sectional online survey study, we applied a German version of the COVID Stress Scales (CSS) and standard psychological questionnaires to systematically identify the complex interplay between stress sensitivity, gender, and personality in the modulation of quality of life and mental health in the German population (*N* = 1774; age ≥ 16 years). A CSS-based cluster analysis revealed two clusters characterized by higher and lower stress levels. Study participants in each cluster differed significantly with respect to neuroticism, extraversion, agreeableness, quality of life, depression, and anxiety. Females were significantly overrepresented in the higher stress cluster, while there was an overrepresentation of males in the lower stress cluster. Neuroticism was identified as a risk factor and extraversion as a protective factor for enhanced pandemic-related stress responses. For the first time our data show a taxonomy of factors, which modulate pandemic-related stress sensitivity and warrant consideration as key indicators of quality of life and psychological distress during the COVID-19 pandemic. We suggest that our data may advise governmental regulation of pandemic-related public health measures, to optimize quality of life and psychological health in different groups of the population.

## 1. Introduction

On the 30^th^ of January 2020, the World Health Organization (WHO) declared the coronavirus disease (COVID-19) outbreak a health emergency of international concern. The COVID-19 pandemic was and still is an enormous challenge for the world, impacting societies in various ways. Social isolation, fear of virus transmission, economic uncertainty, physical discomfort, and the daily deluge of news regarding the numbers of infections and related deaths negatively affect the quality of private, social, and occupational life (1–3). It has repeatedly been shown that both psychological health and quality of life have been negatively affected by the pandemic (4). Vindegaard and Benros (5) reported lower psychological well-being and higher levels of anxiety and depression in the general population. According to Xiong et al. (4), female gender, previous mental or physical illness, socioeconomic status (e.g., unemployment), age, and COVID-19 infections in relatives need to be considered as risk factors for the development of psychological distress and reduced quality of life during the pandemic.

Stress-related experiences during the COVID-19 pandemic are reaching a new level. For example, recent studies found that 25–50% of the German population suffers from symptoms related to stress and emotional distress (5–9). Some authors also report severe stress-related posttraumatic stress symptoms in COVID-19 infected patients (10). Given that half of the German population suffers symptoms of pandemic-related stress (5–9), one needs to consider that the other half must possess specific abilities that are protective against stress symptoms. Risk and protective factors for the development of stress symptoms have not been systematically investigated. Whereas Taylor et al. (11) reported that there is little evidence that personality traits influence COVID-related threat beliefs in adults in voluntary self-isolation, there is some evidence that personality characteristics need to be considered as factors in cognitive and behavioral responses to stress (12). For example, Liu et al. (13) showed that neuroticism might negatively influence pandemic-related stress perception. Some authors have also demonstrated that people with higher extraversion show increased stress reaction during the COVID-19 pandemic (13, 14), while Shokrkon and Nicoladis (15) reported that extraversion is related to better pandemic-related mental health.

A model that has proven particularly useful in explaining the effects of stress in crisis situations like the COVID-19 pandemic is the *Transactional Model of Stress and Coping* by Lazarus and Folkman [*TSC*; (16–18)]. This model posits that stress is a result of the relationship between individuals and their environment, and that sociodemographic factors such as age, gender, socioeconomic status may influence the type and intensity of experienced stress. The model also states that individual perceptions of stressors and their appraisal as either threats or challenges determine the degree of stress experiences. In particular, factors such as knowledge, beliefs, and emotions can influence the individual perception and appraisal of stressors. Furthermore, the TSC suggests that individual coping strategies and resources such as social support and individual skills and competencies play an important role in the processing of stress experiences (16–18). In a pandemic, risk and protective factors related to stress can be influenced by the interaction of individual factors, perceptions, coping strategies, and resources. Personality traits, general health, anxiety, depression, obsessive–compulsive symptoms (OCS) as well as demographic parameters may act as key moderators of stress experiences. Personality traits influence the perception and evaluation of stressors. A low general health status and high levels of anxiety and depression can also lead to a higher sensitivity for stressors and less efficient coping strategies. OCS can itself be seen as a strategy of stress coping by attempting to control situations and reduce uncertainty. At the same time, the symptoms themselves also act as stressors in that they may severely impair everyday living.

Additional contextual factors may be integrated into the theoretical framework of the TSC. Applicable factors are included in the *COVID Stress Scales* (*CSS*) developed and validated by Taylor et al. (19) in population-representative Canadian and North-American samples to better understand and assess pandemic-related distress. A stable 5-factor solution was identified: danger and contamination fears, fear about economic consequences, xenophobia, compulsive checking and reassurance seeking, and traumatic stress symptoms. Subscales were intercorrelated, providing evidence of a COVID Stress Syndrome. The CSS has been translated into 26 languages to date (20–24), validated for the German population (25) and become a gold standard in the assessment of COVID-related stress.

Although numerous studies concerning stress and psychological health during the COVID-19 pandemic have been published, a systematic concept of the interrelationship between the relevant facets is still lacking. Knowledge of the interrelation of specific personality traits, psychological symptoms, pandemic-related stress reactions, and quality of life can provide more detailed information on the taxonomy of individual behavior during the pandemic. In our online survey study, we applied a German version of the CSS of Taylor et al. (19, 25) and standard psychological questionnaires to identify the interplay between stress, personality, and behavior in the German population. We expected that (i) distinct populational clusters related to the amount of stress and strain during the COVID-19 pandemic can be extracted from the data, (ii) that demographic and personality characteristics act as key features in the definition of clusters, and (iii) that quality of life is lowest during the pandemic in population clusters with high levels of stress and strain.

## 2. Methods

### 2.1. Study design

The Psychological Institute of the Johannes Gutenberg-University Mainz, the Faculty of Health of Witten/Herdecke University, and the Psychotrauma Center of the Hospital of the German Armed Forces Berlin conducted the longitudinal online survey entitled “Stress and Strain during the COVID-19 Pandemic” in the German population from August 2020 to June 2021. For each participant, the survey scheduled two time points of measurement 8 weeks apart between August 2020 to June 2021. The timepoint of inclusion in the study was the first response to the questionnaire. Completion of the questionnaire at the second timepoint of measurement was the end of study participation and was carried out 8 weeks after the beginning of study participation. In the case of consent from a participant, the follow-up questionnaire was sent automatically *via* e-mail. The present work analyzes exclusively cross-sectional data of the first timepoint of measurement of this longitudinal study.

### 2.2. Sampling

Participants were invited by flyers, regional and national press releases, e-mail distribution lists, and social media. Inclusion criterium was an age ≥ 16 years. A reimbursement was offered by the opportunity of entering a competition to gain 25 € vouchers for online shopping for participation at the first and second time point of measurement. All included participants gave written informed consent to take part in the study. The study complies with the recommendations of the World Medical Association published in the current version of the Declaration of Helsinki and was approved by the ethics committee of the Psychological Institute of the Johannes Gutenberg-University Mainz.

### 2.3. Sample

Participation in the study was tied to the questionnaires being completed in full. This was ensured by the fact that in the survey software SoSci Survey^1^ it was set by default that the survey could only be ended when the questionnaires were completely filled in. This excluded the possibility of missing data points in the data set. The number of valid participants for the first timepoint of measurement was *N* = 1964. *N* = 118 of these participants did not explicitly indicate informed consent to participate in the study. Moreover, 72 duplicate data sets were detected. The resulting *N* = 190 inaccurate data sets were excluded from statistical analyzes. The final sample included a total of *N* = 1774 adults (females: *N* = 1269; males: *N* = 497; diverse: *N* = 8) with an age range from 16 to 85 years (*M* = 41 years, SD = 14). The sample is representative only for the higher educated German population, with an overrepresentation of females. Table 1 summarizes the sociodemographic characteristics of the sample.

**Table 1:** Summary of sample demographic characteristics.

		Frequency	Percent (%)	Minimum	Maximum	*M*	SD	Mdn	Modus
Age				16	85	41.23	14	40	27
Sex(*N* = 1766)	Male	497	28						
	Female	1269	72						
Marital status (*N* = 1774)	Married/in a relationship	1258	71						
	Single	377	21						
	Widowed	27	2						
	Divorced	112	6						
Children (*N* = 1774)	Yes	944	53						
	No	830	47						
	Number of children			0	7	2	1	2	2
Education degree (*N* = 1774)	Still in School	28	2						
	School finished without graduation	47	3						
	Secondary−/elementary school diploma	224	13						
	Secondary school leaving certificate (Mittlere Reife)	11	1						
	Polytechnic high school	141	8						
	Advanced technical college	387	22						
	High school diploma	907	51						
	University degree	29	2						
Employment (*N* = 1774)	Pupil	37	2						
	Apprentice	17	1						
	Student	269	15						
	Employee	875	49						
	Public servant	154	9						
	Self employed	108	6						
	Unemployed/Jobseeker	31	2						
	Pensioner	112	6						
	Housewife/Houseman	41	2						
	On parental leave	37	2						
	Other	93	5						
Occupational situation in connection with the pandemic (*N* = 1774)	No change	749	42						
	Occasionally home office	261	15						
	Mainly home office	326	18						
	Completely home office	262	15						
	Short-time work	56	3						
	Currently exempted	37	2						
	Financial benefits to support the self-employed	14	1						
	Other financial support	69	4						
Physical illness (*N* = 1755)	No	1314	74						
	Yes	441	25						
Mental illness (*N* = 1764)	No	1539	87						
	Yes	225	13						

### 2.4. Procedure

#### 2.4.1. COVID stress scales

Data were acquired using the online platform SoSci Survey (see text footnote 1). Questionnaires included a German adaptation of the CSS (25), which consists of 36 items. The translation-back-translation process followed the guidelines for translating foreign language self-report measures. The original English items were transformed into German by Professors Jun.-Prof. Dr. S. M. Jungmann and Univ.-Prof. Dr. M. Witthöft and then back into English by a professional bilingual translator whose mother tongue is English. Although there were slight variations in wording between the two English versions (e.g., “keep me safe” vs. “protect me,” “mail handlers” vs. “postman,” “professionals” vs. “experts”), these differences were retained due to the preservation of the overall content. The German version consisted of 36 items, organized into six items per scale, and responses were recorded on a 5-point Likert scale ranging from 0 (not at all) to 4 (extremely), except for the Traumatic Stress and Compulsive behavior/reassurance related to frequency scales, which were answered on a scale ranging from 0 (never) to 4 (almost always). For the final German version of the CSS, see https://coronaphobia.org/professional-resources/ (25) The initial CSS construct includes 6 domains: COVID danger, COVID contamination fear, COVID fear about socioeconomic consequences, COVID xenophobia, COVID traumatic stress symptoms, and COVID compulsive checking and reassurance seeking related to the COVID-19 pandemic. In the Canadian validation of the CSS, Taylor et al. (19) found that the subscales COVID danger and COVID contamination fear loaded on the same factor, suggesting a 5-factor CSS model. However, the authors retained the 12 items of the two domains in the CSS, such that the option of assessing COVID danger separately from COVID contamination fear remains methodologically and in terms of content justified. Since we aimed at assessing the German COVID situation in most detail, we separated the two scales in our cluster analysis approach. The CSS was shown to be reliable and valid in a nonclinical German population (ranging from ω = 0.70–0.94 and r_tt_ = 0.62–0.82; 25).

#### 2.4.2. Psychological standard test procedures

We applied a short form of the World Health Organization Quality of Life Assessment (WHOQOL-BREF; 26), the Patient Health Questionnaire-4 (PHQ-4; 27), the Obsessive Compulsive Inventory-Revised (OCI-R; 28), and the 10-item Big Five Inventory (BFI-10; 29).

The WHOQOL-BREF is a questionnaire developed by the World Health Organization for the subjective assessment of one’s quality of life (26). The questionnaire measures a self-report health status across the four health domains Physical Health (Physical), Psychological (Mental), Social Relationships (Social relations), and Environment. Respondents indicate their perceived health status for each item on a 5-point Likert scale. The questionnaire includes three negatively pooled items. The internal consistency of the WHOQOL-BREF ranges from *α* = 0.57 to *α* = 0.88 (27, 28).

The PHQ-4 is a self-report questionnaire on depression and anxiety (29). Participants determine the severity of symptoms for the past 2 weeks on a 4-point Likert scale. Validity and reliability was demonstrated in non-clinical and clinical samples (30). In this study, the internal consistency of the PHQ-4 McDonald’s omega was *ω* = 0.88. (25).

The OCI-R measures symptoms of obsessive–compulsive disorder (31). The questionnaire consists of 18 items across the six subscales *washing, checking, ordering, obsessing, hoarding*, and *neutralizing*. Respondents indicate on a 5-point Likert scale the individual intensity related to the symptoms described by each item for the past month. The OCI-R was shown to be reliable and valid in nonclinical and clinical samples (31, 32). In this study, the internal consistency of the OCI-R was *ω* = 0.88 (25).

The BFI-10 measures personality traits based on the five-factor model (33). The questionnaire consists of 10 items. Each of the five dimensions (extraversion, agreeableness, conscientiousness, neuroticism, openness) is represented by two items of the questionnaire (one positive and one negative pooled item). The items are answered using a 5-point rating scale. Reliability and validity of the BFI-10 was demonstrated based on a population-representative sample (33).

#### 2.4.3. Further measures

Furthermore, a self-constructed questionnaire on cognitive models of a pandemic was integrated into the survey (6 items, e.g., “I think the spread of the virus resulted from an “accident” in a genetics laboratory,” “I think the spread of the virus is bad for people’s lives, but good for the environment”). Items of this questionnaire had to be rated on a 5-point Likert scale ranging from “not at all” to “extremely.” In addition to the questionnaire data, the online survey collected sociodemographic data on age, sex, highest educational attainment, marital status, profession, occupational situation related to the pandemic, health status (physical and/or mental illness), own coronavirus infection or infections of relatives, friends etc., quarantine situations due to coronavirus infection, and one’s occupational situation during the COVID pandemic.

### 2.5. Statistical analysis

To identify classes from the total sum score of the CSS, a two-step cluster analysis was performed. This cluster analysis is particularly suitable for large data sets. It allows for extraction of the optimum number of clusters from the data in 98% of cases according to Chiu et al. (34). Moreover, it is an exact and powerful procedure even in the presence of overlap for metric variables (35). Demographic characteristic variables were not included in the cluster analysis to avoid mixed-scaled grouping, which has the disadvantage of giving higher weights to differences in categorical variables such that the clustering procedure could be biased. This effect is amplified, when a large difference in the group size of a categorical classification (36) occurs. This is the case in our data set including considerably more females than males. For the cluster analysis, a log-likelihood measure was selected as the distance measure. Schwarz’s BIC was chosen for automatic clustering. Cases were randomly ordered, and variables were standardized by cluster analysis. A noise cluster of 5% was applied in the clustering procedure to eliminate outliers. Cluster divisions resulting from the analysis were stored in the dataset using cluster variables. These provided the basis for subsequent statistical post-hoc analyzes.

For testing the factor solution of the CSS total sum score and to extract the relevance of CSS subscales for cluster classification, a second two-step cluster analysis was performed with the CSS subscales as variables. For automatic clustering, the cluster criterion BIC according to Schwarz (37) and the log-likelihood measure were used as distance measures. A noise cluster of 5% was applied to eliminate outliers. To test the factor solutions of the two cluster procedures for independence, a Chi^2^-test was performed between the cluster variables of the two cluster solutions.

To identify additional potentially discriminating characteristics between clusters, a statistical post-hoc comparison of scores on the WHOQOL-BREF, OCI-R, PHQ-4, BFI-10, and cognitive model questionnaire, split by cluster variables of the sum score of the CSS, was performed using T-tests (Welch-tests were applied in cases of variables without variance homogeneity). The level of statistical significance for separate group comparisons for each single variable was set to *p* < 0.05. To test putative differences in sociodemographic characteristics (age, gender, occupational situation, marital status, children, mental illness, physical illness, educational attainment, employment, quarantine due to Corona infection, own Corona infection, Corona infection in close environment, Coronavirus infection in professional environment, vacations) between the two clusters for independence, a contingency analysis was performed using a Monte Carlo Simulation (given an expected cell frequency < 5). To detect a potential overrepresentation of sociodemographic characteristics between clusters, a comparison of column proportions was applied using Z-tests, adjusted for multiple comparisons. All statistical analyzes were implemented using SPSS, version 27 (Statistical Package for the Social Sciences; https://www.ibm.com/de-de/products/spss-statistics).

## 3. Results

### 3.1. Identification of clusters

#### 3.1.1. COVID stress scales total Sum score cluster analysis

Cluster analysis of the CSS total score resulted in two clusters with sufficient scores for cluster cohesion and cluster separation. The silhouette measure of 0.7 corresponds to an acceptable to strong indication of cluster structure according to Kaufman and Rousseeuw (28). The size ratio of the two clusters is a value of 1.49. Cluster 1 contains 1049 (~60% of the sample) individuals with a mean CSS total score of *M* = 15.90 (SD = 7.63). Cluster 2 includes 702 (~40% of the sample) individuals with a mean CSS total sum score of *M* = 42.84 (SD = 12.18). Based on the mean scores of each cluster, a designation of Cluster 1 as “Lower CSS” and Cluster 2 as “Higher CSS” was made.

#### 3.1.2. COVID stress scales subscales cluster analysis

The clustering procedure of the CSS subscales also resulted in a factor solution of two clusters. The procedure yielded a moderate result for cluster cohesion and cluster separation, which corresponds to a low indication of a cluster structure with a silhouette measure of 0.4. The size ratio of the two clusters has a value of 1.62, with Cluster 1 containing 1081 (~62% of the sample) individuals and Cluster 2 containing 666 (~38% of the sample) individuals. Mean scores of subscales in Cluster 1 were lower than the mean scores of the same subscales in Cluster 2, and the subscales in each of the two clusters had the same gradation in importance of the subscales as predictors of cluster classification. Accordingly, Cluster 1 was named “Lower CSS subscales,” and Cluster 2 was named “Higher CSS subscales.” There was minor overlap between the clusters for the Xenophobia and Traumatic Stress subscales and medium overlap for the Compulsive Checking and Socioeconomic Consequence subscales. Table 2 shows the descriptive scores for each subscale of the CSS, split by cluster assignment and graded by predictor influence.

**Table 2:** Mean values and standard deviations of the subscales of the CSS, divided by cluster classification, sorted by predictor weighting.

	Cluster 1 Lower CSS subscales (*n* = 1081)		Cluster 2 Higher CSS subscales (*n* = 666)		Importance (predictor weighting)
	*M*	SD	*M*	SD	
Danger (domain)	5.47	3.87	12.75	3.92	1
Contamination (domain)	3.58	2.77	9.36	4.09	0.7
Xenophobia	2.58	2.82	7.53	4.72	0.4
Traumatic stress	1.33	1.95	4.96	4.16	0.3
Obsessive control	1.72	2.08	4.55	3.49	0.2
Socioeconomic consequence	1.22	2.01	3.70	3.68	0.2

CSS = Covid Stress Scale. The subscales can range from 0 (no symptoms) to 24 (severe symptoms); Domains Danger and Contamination = Subscale “COVID Danger and Contamination fears,” Xenophobia = Subscale “COVID Xenophobia,” Traumatic stress = Subscale “COVID traumatic Stress symptoms,” Obsessive control = Subscale “COVID compulsive checking and reassurance seeking,” Socioeconomic consequence = Subscale “COVID fears about socioeconomic Consequences”; *N* = 1774.

The Chi^2^ test between cluster divisions showed a significant relationship between cluster divisions of cluster procedures of both the total score and the subscales of the CSS (X^2^(1) = 1425, *p* < 0.001, φ = − 0.90). Due to the comparable size ratio and the significant correlation with a high effect size between the cluster solutions of the two cluster procedures, it can be expected that cluster division is stable across factor solutions of the two cluster procedures.

### 3.2. Questionnaires

**Figure 1:**
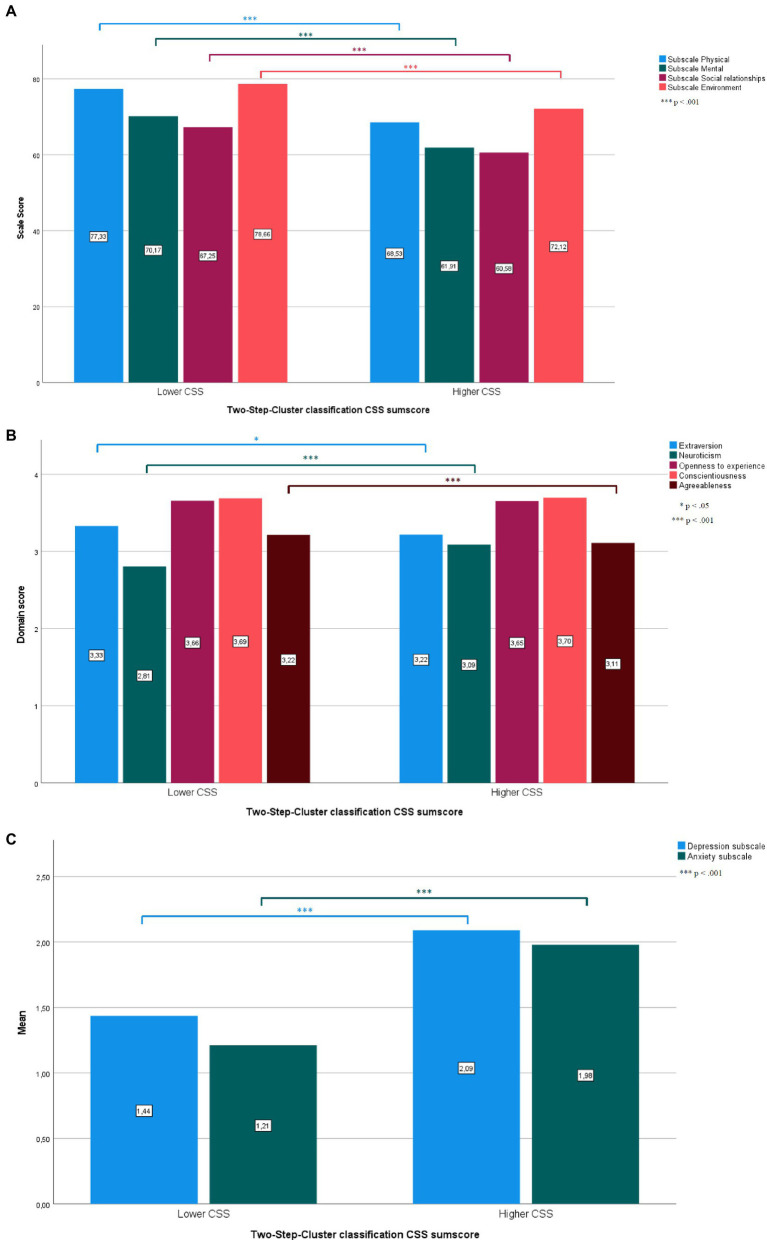
Significant and non-significant differences between the higher and lower CSS clusters in the WHOQoL-BREF **(A)**, BFI-10 **(B)**, and PHQ-4 **(C)**. Detailed statistical data are given in Table 3. **(A)** Scale values of the WHOQoL-BREF split by Cluster classification. **(B)** Domain Scores of the BFI-10 split by Cluster classification. **(C)** Mean values of the PHQ-4 split by Cluster classification.

T-tests indicated that the lower (compared to the higher) CSS cluster was characterized by significantly lower scores on the neuroticism dimension as well as higher scores on the extraversion and agreeableness dimensions of the BFI-10. Moreover, the lower CSS stress cluster scored significantly lower on the depression and anxiety dimensions of the PHQ-4. The higher (compared to the lower) CSS cluster showed significantly lower scores on the physical social relationships and environment subscales of the WHOQOL-BREF, as well as significantly higher scores in the washing, obsessive–compulsive ideation, hoarding, ordering, controlling, and thought neutralizing subscales of the OCI-R. Furthermore, the subjects in higher CSS cluster were significantly older and scored higher in the pandemic background questionnaire than subjects in the lower CSS cluster. Table 3 gives detailed statistical information on questionnaire data divided by cluster classification. Figure 1 illustrates WHOQL-BREF, BFI-10, and PHQ-4 data divided by cluster classification.

**Table 3:** Means and standard deviations of the BFI-10, WHOQoL, OCI-R, and PHQ-4, age, and Pandemic background questionnaire, divided by cluster classification.

		Cluster 1 Lower CSS Cluster (*n* = 1049)		Cluster 2 Higher CSS Cluster (*n* = 702)		*t* (1749)	*p*_two-sided_	Cohen’s d
		*M*	SD	*M*	SD			
BFI-10	Extraversion	3.33*	0.98	3.22	1.01	−2.28	0.0230	−0.111
	Neuroticism	2.81***	0.97	3.09	0.94	6.05	<0.001	0.295
	Openness to experience	3.66	0.94	3.65	0.96	−0.06	0.949	0.003
	Conscientiousness	3.69	0.80	3.70	0.77	0.22	0.829	0.011
	Agreeableness	3.22***	0.74	3.11	0.78	−2.91	0.004	−0.142
WHOQoL-BREF	Physical	77.33	14.75	68.53***	17.18	−11.09	<0.001	−0.557
	Mental	70.17	17.73	61.91***	17.91	−9.52	<0.001	−0.464
	Social relations	67.25	20.16	60.58***	21.47	−6.53	<0.001	−0.322
	Environment	78.66	12.79	72.12***	13.98	−9.91	<0.001	−0.492
OCI-R	Washing	0.84	1.42	1.97***	2.16	12.22	<0.001	0.645
	obsessive–compulsive ideation	1.69	2.15	2.90***	2.59	10.28	<0.001	0.520
	Hoarding	2.24	2.23	2.88***	2.52	5.52	<0.001	0.276
	Order	2.26	2.53	3.32***	2.85	7.96	<0.001	0.397
	Controlling	1.74	1.99	2.73***	2.43	8.96	<0.001	0.454
	Thought neutralization	0.78	1.39	1.30***	1.90	6.26	<0.001	0.324
PHQ4	Depression	1.44***	1.40	2.09	1.49	9.32	<0.001	0.455
	Anxiety	1.21***	1.41	1.98	1.66	10.07	<0.001	0.507
Age		40.57	13.89	42.06*	14.40	2.18	0.030	0.106
Pandemic background questionnaire		9.88	3.20	10.22*	3.07	2.25	0.024	0.110

BFI-10, 10-item Big Five Inventory; WHOQOL-BREF, short form of the World Health Organization Quality of Life Assessment; OCI-R, Obsessive Compulsive Inventory-Revised; PHQ-4, Patient Health Questionnaire. *N* = 1774. * *p* two-sided < 0.05 *** *p* two-sided < 0.001.

### 3.3. Cluster demographics

There was a significant relationship between cluster classification and gender [*X*^2^(1) = 4.40, *p* < 0.05, φ = 0.05], the variables mental illness [*X*^2^(1) = 7.12, *p* < 0.01, φ = − 0.06], physical illness, [*X*^2^(1) = 43.25, *p* < 0.001, φ = − 0.16], educational attainment, [*X*^2^(7) = 51.61, *p* < 0.001, *V* = 0.17], employment, [*X*^2^(10) = 38.89, *p* = 0.001, *V* = 0.13], occupational situation during the pandemic [*X*^2^(7) = 17.40, *p* < 0.05, *V* = 0.10], the variable “quarantine due to Corona infection” [*X*^2^(2) = 7.57, *p* < 0.05, *V* = 0.06], and the variable “Coronavirus infection in occupational environment” [*X*^2^(7) = 5.44, *p* < 0.05, *V* = 0.06]. Table 4 shows the cross tabulation of column proportion tests for sociodemographic characteristics between lower CSS and higher CSS, adjusted for multiple comparisons. Women, people with physical or mental illness, people with secondary school, junior high school and technical college degrees, pensioners, housewives or housemen, people who had been in quarantine in the previous weeks before the survey, participants who knew about infected people in their occupational environment, and persons who received other financial support were significantly overrepresented in the higher CSS cluster (all *p* < 0.05). Males, people without physical or mental illness, participants with a university degree, students, and participants who did not knew about infected people in their occupational environment were significantly overrepresented in the lower CSS cluster (all *p* < 0.05). For the variables “marital status,” “children,” “Corona infection,” “Corona infection in close environment,” and “vacations” no significant relationship to cluster classification was detected (all *p* > 0.05).

**Table 4:** Cross tabulation of column proportion tests for sociodemographic characteristics between lower CSS and higher CSS.

		Cluster 1 lower CSS (*n* = 1049)	Cluster 2 higher CSS (*n* = 702)
		Number as columns (%)	Number as columns (%)
Psychiatric illment	No	89_a_	85
	Yes	11	15_b_
Physical illness	No	81a	67
	Yes	19	33b
Gender	Male	30_a_	25
	Female	70	75_b_
Education degree	Pupil	1	2
	School finished without graduation	0	0
	Secondary−/elementary school diploma	2	4_b_
	Secondary school leaving certificate (Mittlere Reife)	11	16_b_
	Polytechnic high school	0	1
	Advanced technical college	6	10
	High school diploma	21	23
	University degree	58_a_	42
	Other high school diploma	1	2
Employment status	Pupil	2	3
	Apprentice	1	1
	Student	18_a_	11
	Employee	50	49
	Public servant	9	9
	Self employed	7	5
	Unemployed / Jobseeker	1	2
	Pensioner	5	8_b_
	Housewife / Houseman	1	4_b_
	On parental leave	2	3
	Other	5	6
Quarantine due to Corona infection	No	87	83
	Yes, currently	1	1
	Yes, in weeks past	12	16_b_
Coronavirus infection: Yes, myself.	No	98	97
	Yes	2	3
Coronavirus infection: Yes, someone in my personal environment.	No	58	56
	Yes	42	44
Coronavirus infection: Yes, someone in my professional environment.	No	70_a_	65
	Yes	30	35_b_
Vacation	No	55	51
	Yes	45	49
Occupational situation in connection with the pandemic	No change	43	40
	Occasionally home office	15	15
	Mainly home office	19	18
	Completely home office	15	15
	Short-time work	3	3
	Currently exempted	2	3
	Financial benefits to support the self-employed	1	1
	Other financial support	2	6_b_
Children	Yes	54	52
	No	46	48
Marital status	Married/partnership	71	72
	Single	22	21
	Widowed	1	2
	Divorced	6	6

A The columns show the percentage distribution of each item of a variable within each cluster; The subscripts indicate which proportion of the respective item is significantly overrepresented in which cluster; *N* = 1774.

*a higher proportion in the lower CSS Cluster than in the higher CSS Cluster; comparison of column proportions with correction for multiple comparisons; *p* < 0.05.

*b higher proportion in the higher CSS Cluster than in the lower CSS Cluster; comparison of column proportions with correction for multiple comparisons; *p* < 0.05.

## 4. Discussion

In this study, we identified two CSS-based clusters in the German population characterized by higher and lower stress levels during the COVID-19 pandemic. Clusters mainly differed significantly with respect to neuroticism, extraversion, agreeableness, quality of life, symptoms of obsessive–compulsive disorder, depression, anxiety, and cognitive models of a pandemic. Females, people with mental or physical illness, secondary school, junior high school, and technical college degrees, retirees and housewives/−men, people dependent on financial support, people who had been in quarantine in the previous weeks before the survey, and participants who knew about infected people in their occupational environment were significantly overrepresented in the higher stress cluster. Our data show for the first time the complex implications of pandemic-related stress sensitivity in the German population, which may act as key modulators of quality of life and psychological distress during the COVID-19 pandemic. Importantly, we also demonstrate relationships of modulating effects of stress-sensitivity with personality and gender. We propose that our data may advise governmental regulation of pandemic aiming at the optimization of quality of life and psychological health in different groups of the population.

### 4.1. Risk factors according to the cluster analysis approach

Around 36–40% of people experienced heightened stress and strain during the pandemic. This is consistent with prior research showing that 25–50% of Germans exhibited symptoms related to stress and emotional distress during COVID-19 (5–9). However, our study reveals that pandemic-related stress and strain caused significant psychosocial distress in almost half of the German population from the beginning of the first wave to the end of the third. Although CSS scores in the higher cluster were moderate, the results showed a strong impairment in different dimensions of quality of life (see Quality of life and mental health). Our study focused primarily on pandemic-related stress. Even moderate CSS scores add a substantial pandemic-related burden to the individual’s already complex daily stress, leading to chronic stress. Chronic stress may cause physiological changes and health problems (38, 39) and further psychosocial effects on the individual and society. However, moderate scores indicate that most people can establish efficient adaptation processes to prevent clinically relevant physiological and mental illnesses from developing despite the pandemic’s significant demands on the population. Cluster classification indicates a gradual relevance of pandemic related triggers of additional stress on the population. Worry about the dangerousness of COVID-19 represents the highest stress burden of the pandemic. This is supported by the study of Taylor et al. (3), who also used the CSS and identified the dangerousness of COVID-19 as a central feature of the Covid-19 Syndrome in Canadian and American people. The finding that the CSS subscale “Worry about the dangerousness of COVID-19” (COVID danger) represents the highest stressor, and that the CSS subscales “fear of contamination” (COVID contamination fear) and “danger of contagion by foreigners” (COVID xenophobia) act as key factors for the accumulation of stress during the pandemic fits into the secondary stage of appraisal of the TSC. During secondary appraisal, individuals evaluate whether a stressor represents a harmful threat or a challenge. In the case of the COVID-19 pandemic, above stressors are subjectively interpreted as threats to an individual’s health and the convincement that life will continue as before. Worry in the face of these threats can be influenced by various factors, including personal beliefs, cultural background, and exposure to information about the pandemic by media. Moreover, individuals who have pre-existing health problems or belong to high-risk groups may experience higher worry about the potential danger of COVID-19. In our study, trauma experiences, obsessive control, and socioeconomic consequences of the pandemic turned out to be less relevant for pandemic-related stress accumulation (COVID traumatic stress symptoms, COVID compulsive checking and reassurance seeking related to the COVID-19 pandemic and COVID fear about socioeconomic consequences). Given these data, existential health-related fear can be considered as the most important target for qualified information of heterogeneous groups of the population, psychotherapeutic interventions, and support of the population for ameliorating the pandemic-related stress accumulation. Given the high concerns of contagion by foreigners, xenophobia also needs to be considered in successfully managing pandemic-related stress in the population. This perceived threat related to foreigners can be explained by unconscious distal defense mechanisms. These may come into play when individuals are confronted with their mortality. They then tend to show national identification and hostility toward outgroups (40). For example, a prototypical distal defense mechanism is the designation of COVID-19 as a “Chinese virus.” This interpretation is also supported by multidisciplinary neuroscience studies showing that stress favors the release of oxytocin, which in turn contributes on the behavioral level to the occurrence of a “tend and defend” reaction in favor of the ingroup (41–45).

### 4.2. Further protective and risk factors of pandemic-related stress sensitivity

Analyzes of cluster-specific socio-demographic characteristics revealed protective and risk factors for stress and strain in the pandemic. These are in particular related to gender, personality, education, quarantine, current mental and/or physical illnesses and older age.

#### 4.2.1. Gender

The influence of gender on the experience of stress during the pandemic emerges from a complex interaction of a wide variety of factors. In our study, we identified female gender as a risk factor for increased pandemic stress and strain. Stress arises from the relationship between individuals and their environment. Individual sociodemographic differences such as gender may influence the type and intensity of perceived stress (16–18). It is known that women use other strategies to cope with stress than men do. Such gender-related differences may be due to social and cultural factors, for example, gender-specific role expectations, as well as biological factors such as hormones and genetics. These factors are likely to contribute to a higher stress levels and reduced capacities of efficient stress coping strategies in females. Our finding is in accordance with the narrative review by Almeida et al. (46) and the systematic review by Xiong et al. (4), who concordantly reported that female gender is a risk factor of distress during the pandemic and that women who are pregnant, postpartum, miscarrying, or experiencing intimate partner violence are at high risk for developing mental health problems during the pandemic. It is known that males and females differ in their perception of stressors and coping with perceived stress. Females more frequently report health-related and family events as sources of stress than do males. In contrast, men perceive themselves as mainly stressed by financial and work-related events (47, 48). Given these data, one explanation for the elevated stress levels in women during the pandemic may be that they anticipate and ruminate more intensely than men about putative negative health consequences for themselves and their family.

Gender differences also need to be considered with respect to stress coping strategies. Some older studies indicate that females tend to use social support and help-seeking as primary strategies to cope with stress (49, 50). A lockdown including drastic restrictions on social contact during the pandemic may thus prevent females from using their preferred stress coping strategies, leading to the enhancement of their subjective feelings of stress. Moreover, in our society females face specific burdens that have become more virulent during the pandemic. For example, unpaid care work has been reported as a special stress factor–especially for the female gender (51, 52). Care work is the main basis of everyday life (e.g., childcare, household, etc.), but often remains unnoticed and acts as an invisible stressor. Since this work is predominantly shouldered by women, the invisible stressor exacerbated for females by the pandemic (52).

#### 4.2.2. Personality

Importantly, our data also indicate that subjects in the cluster with lower stress had higher scores in extraversion, suggesting that this personality trait protects against pandemic-related stress accumulation. Available studies show heterogeneous results concerning this issue. This heterogeneity of data results at least in part from data collection during distinct time windows in the pandemic. For the severe first COVID-19 wave Engert et al. (14) demonstrated that extraversion is related to higher cortisol levels, indicating a more intense stress response of the organism. During the same time frame, Liu et al. (13) also showed in a Canadian sample, that higher extraversion is associated with higher general stress levels in the pandemic. In the period after the first wave, Getzmann et al. (53) reported that extraversion had no influence on stress in the population. Bellingtier et al. (54) reported for the third wave of the pandemic that only the sociability facet of extraversion was related to higher stress levels. In contrast, the assertiveness facet was found to act as a protective factor against stress. Our survey had a more extended time frame than all previous studies. We started data collection after the first wave of the pandemic and finished the study after the third wave of the pandemic. Across these distinct phases of the COVID-19 pandemic, extroverted people showed lower stress levels than non-extroverted people. Most likely, people developed efficient coping strategies during the long time frames with pandemic-related socializing restrictions, in particular, in summer 2020 where we started data collection. Moreover, extroverted people may have acquired stress resilience during phases with relaxed social restrictions and then profited from resilience in following phases with enhanced restrictions (55). This interpretation is in line with the reports for the first wave of both Engert et al. and Liu et al. (13, 14). It also fits with the data of Getzmann et al. (53) and does not contradict the findings of Bellingtier et al. (54). Our arguments are also supported by a study of Shokrkon and Nicoladis (15), who demonstrated a positive effect of extraversion on overall mental health during the COVID-19 pandemic. Besides extraversion, we identified agreeableness as a protective factor against stress and strain during the pandemic. This result is in line with previous reports of the protective impact of this personality trait against stress in general and with respect to the pandemic (56–58). Interestingly, Gori et al. (57) demonstrated by a mediation analysis, that agreeableness is only indirectly involved in protection against stress during the pandemic, due to its negative association with maladaptive defense mechanisms. While extraversion may actively trigger efficient coping strategies, agreeableness may be rather passively involved in defense mechanisms, which actively dominate stress-related coping behavior during the pandemic. In line with the TSC, individuals with higher levels of extraversion and agreeableness may be better equipped to handle altered living conditions and restrictions during a pandemic. Extraverted individuals typically possess a stable and extended social network, are able to fulfill their social desires by virtual communication, and search continuously for novel ways to experience social joy. Doing so, they are supported by their positive attitude and optimistic thinking. Individuals with higher levels of agreeableness typically possess enhanced empathy and aim at avoiding conflicts. Accordingly, they also tend to follow rules set by society and authorities. Adherence to rules, which are perceived as a form of security by people with high levels of agreeableness, could therefore reduces stress.

The results of our study demonstrate that the personality trait neuroticism acts as a key factor in the identification of risk groups for elevated stress in the Covid-19 pandemic. Neuroticism has been linked by previous studies to elevated stress levels and depression in the pandemic (13, 14, 54, 59, 60). Furthermore, Liu et al. (13) and Zager Kocjan et al. (61) showed that neuroticism functions as a predictor of diminished adaptive behavior and may lead to reduced perceived self-efficacy. According to the TSC (62) this finding can be related to secondary appraisal of stressors. Individuals with neuroticism possess few stress coping strategies that could efficiently help in resolving pandemic-related stressful situation and phases. This may reinforce low perceived self-efficacy and the persistence of threat-guided stress reactions. In summary, elevated stress responses may arise from a specific appraisal of stressors and the efficacy of stress coping mechanisms (62). Accordingly, individuals with neuroticism may exhibit an enhanced perception of stressors as uncontrollable threats.

#### 4.2.3. Education

A further modulator of pandemic-related stress is an individual’s level of education. In line with the study of Taylor et al. (3) in Canadian and US samples, we show increased pandemic-related stress levels for groups with lower education in the German population. Given that people with low education are typically exposed to higher levels of stress than people with high education (63), this could be an effect of cumulative stress experience during the pandemic. This interpretation is in good accordance with reports that lower income and reserves causing financial problems during the pandemic were frequently associated with the emergence symptoms of anxiety and depression (64–66). A second explanatory approach relates to the fact that people with lower levels of education are more likely to hold jobs that require face-to-face contact than people with high educational levels (67). This situation increases the probability of getting infected with COVID-19 and may also explain our finding that people who knew about infected people in their occupational environment showed elevated stress levels. We here detected facets of dynamics of interaction between low education and occupation-related stress during the pandemic.

#### 4.2.4. Quarantine

Our result of an overrepresentation of people who have previously been in quarantine in the higher stress cluster is in line with previous research on psychological health in the population during the COVID-19 pandemic. For example, the TMGH-Global COVID-19 Collaborative study (68) demonstrated, that a longer duration of quarantine significantly correlated with higher stress levels. This finding is also in line with the TSC since quarantine itself needs to be considered as a significant source of stress. Quarantine can be appraised as a loss of freedom and a threat to one’s well-being within the secondary evaluation of the stressor.

#### 4.2.5. Illness

Consistent with two studies of Asmundson et al. and a study of Xiong et al. (4, 69, 70) we identified previous or current mental and/or physical illnesses as risk factors for increased stress in the pandemic. Physical pre-existing conditions can increase the risk of experiencing severe complications in the event of a COVID-19 infection. This health threat can lead to an increased perception of stress and influence the behavior of affected individuals, for example, by isolating themselves due severe anxiety of contamination. Pre-existing mental diseases or Symptoms such as anxiety disorders, depression and OCS can also increase threat of COVID-19 infection and lead to the avoidance of social contacts. Both pre-existing physical and mental disorders may therefore lead to an enhanced secondary threat evaluation of COVID-19 according to the TSC. Lifetime strain of affected people is too severe for an appraisal of the pandemic as a challenge. Although cluster analysis indicated a lower importance of OCS in the COVID Stress Scale (CSS), our results suggest that individuals showed increased OCS during the pandemic. Specifically, during the COVID-19 pandemic, people exhibited symptoms of compulsive behaviors, such as frequent hand washing or obsessive cleaning of surfaces. These symptoms can be understood as a reaction to the threat posed by the pandemic and the associated uncertainties and changes in daily life. In the TSC, the primary appraisal in this case may be focused on the threat posed by the virus and its potential consequences for health (16–18, 62). The secondary appraisal could then lead to individuals attempting to cope with the threat by performing behaviors such as frequent hand washing or obsessive cleaning of surfaces. Furthermore, increased OCS may suggest that some of the measures recommended by the government, such as the frequent washing of hands, have been effective.

#### 4.2.6. Age

Concerning age, our data diverge from those of Xiong et al. (4). Our results indicate that older age (> 40 years) is a risk factor for higher levels of pandemic-related stress, while Xiong et al. (4) reported younger age (< 40 years) to be a risk factor for enhanced stress levels during the pandemic. The effect of age on stress experience during the pandemic may also depend at least in part on the societal and cultural background of investigated samples. Overall, our data indicate that older age interacts with the variables female gender, low educational level, quarantine experience, previous or current mental and/or physical illnesses in the emergence of pandemic-related high stress levels.

### 4.3. Quality of life

Our data show a severe pandemic-related decline in quality of life in at least half of the German population. These data are in line with previous studies on quality of life during the pandemic (4, 71). Xiong et al. (4) specifically reported increased anxiety, depression, post-traumatic stress, and psychological stress in both eastern and western countries. It is also known that neuroticism is frequently related to a higher risk for the development of depression, anxiety, and obsessive–compulsive behavior (72, 73). Available data well fit with our finding of a close interrelationship between high levels of neuroticism and enhanced pandemic-related stress. Published data on the role of gender for quality of life during the pandemic are heterogeneous. Some authors reported an increased vulnerability for depression and anxiety of females (4, 74, 75), others did not (76, 77). Distinct cultural backgrounds of investigated samples may at least in part account for the heterogeneity of data. For Germany, Abreu et al. (78) demonstrated that women had higher levels of depression, while men showed enhanced aggression in the pandemic. Available data suggest that a modulating role of gender most likely depends on culture, society, population subgroups, and further demographic and historical variables.

Finally, it also needs to be taken into consideration that a biologically based stress sensitivity (e.g., due to variations of gene polymorphisms) may trigger pandemic-related depression and therefore diminish quality of life (79–81). For example, Caspi et al. (79) showed that a polymorphism in the promoter region of serotonin transporter gene modulates resilience against stressful life events with respect to their influence on the incidence of depression.

### 4.4. Limitations

Limitations of the study include the high educational level of the sample and the overrepresentation of women in the sample, as well as the exclusion of children and adolescents under 16 years of age. In terms of statistics, cluster analyzes provide only moderate information on the mode of action and integration of the identified protective and risk factors for pandemic-related stress. Nevertheless, this was the optimal approach for the purpose of this study, as we initially wanted to limit ourselves to an identification of risk factors for stress experience within the pandemic. Regarding the statistical methodology, it should be taken into account that high correlations between the questionnaire results and the parameters of the CSS clustering could lead to an overestimation of the significant differences between the questionnaire results in the individual clusters. To minimize this problem, we ensured that correlations between questionnaire and CSS subscales were either absent or at least low to moderate. It should be noted that the study conducted was an online survey. This type of survey involves various biases that need to be taken into account. Several types of bias can occur in surveys regarding access to the internet, which can affect the accuracy and representativeness of the results. These include the technological divide, where people without access to the internet cannot participate in online surveys and therefore above-average technology-savvy and affluent respondents are captured. Another factor is age bias, where older people have less access to or are less familiar with the internet than younger people. There may also be education bias, where people with lower levels of education have less access to the internet and computer skills. Another important limitation of the study is that the individual measurement points are collected over a longer period of time. This poses several problems in terms of cross-sectional data. The data of the analyzes of this study only contain data on an individual survey of the subjects in the course of the entire study period, without a follow-up survey. However, the condition of these subjects could have changed over the course of the study duration, but was no longer recorded in the study, as only one measurement was taken. Although the data do not allow us to explain exactly whether certain groups of people experienced more or less stress in the course of the pandemic or in different waves, the large number of participants provides a representative sample of the groups of people over the entire course of the measurement period, which allows general statements to be made about the experience of stress and strain. In addition, the data from several different waves were combined in the analysis, which means that we cannot make any statements in this study about the influence of specific measures to contain the pandemic on people’s stress experience. Longitudinal studies with several measurement points are needed to clarify questions in this regard. Furthermore, future studies on pandemic-related stress and mental health in different populations and cultures should include the influence of government regulations during a pandemic as a target variable.

### 4.5. Conclusion

Our data show that pandemic-related stress sensitivity needs to be considered as a key modulator of quality of life and mental health during the COVID-19 pandemic. We specify both protective and risk factors for the development of pandemic-related stress in a non-clinical sample of the German population. We propose that our data may advise governmental regulation of pandemics to optimize quality of life and psychological health in different groups of the population. Societal support and psychological help such as interventions targeting existential health-related fear should be among the central targets of governmental regulation. Finally, psychotherapeutic and medical treatment of citizens suffering from long-term consequences of pandemic-related stress and strain need to be adapted to the complex and rapid changing challenges. To accomplish this adaptation, novel multidisciplinary psychotherapeutic and medical health care structures need to be established. These require both financial and institutional support by the government.

## Data availability statement

The datasets presented in this article are not readily available because it will be used for other publications that are in the process of being analyzed and published. Requests to access the datasets should be directed to Martina.Piefke@uni-wh.de.

## Ethics statement

The studies involving human participants were reviewed and approved by Ethics committee of Psychological Institute of the Johannes Gutenberg-University Mainz. Written informed consent from the participants’ legal guardian/next of kin was not required to participate in this study in accordance with the national legislation and the institutional requirements.

## Author contributions

VN and SJ organized data collection. MP planned, conceptualized, and supervised the study. VN and MP conceptualized the submitted paper and wrote the main parts of the manuscript. VN accomplished statistical data analysis. GW contributed his psychiatric and scientific expertise to the study and was involved in data collection. GA developed the English version of the COVID Stress Scales (CSS) and contributed his scientific expertise. All authors contributed to the article and approved the submitted version.

## Funding

The study was accomplished without funding. The study was financed by the Chair of Neurobiology and Genetics of Behavior of Witten/Herdecke University (Chairholder: MP).

## Conflict of interest

The authors declare that the research was conducted in the absence of any commercial or financial relationships that could be construed as a potential conflict of interest.

## Publisher’s note

All claims expressed in this article are solely those of the authors and do not necessarily represent those of their affiliated organizations, or those of the publisher, the editors and the reviewers. Any product that may be evaluated in this article, or claim that may be made by its manufacturer, is not guaranteed or endorsed by the publisher.

## Footnotes

^1^ https://www.soscisurvey.de.
